# Reproducibility in cheminformatics and computational chemistry research: certainly we can do better than this

**DOI:** 10.1186/1758-2946-5-S1-O4

**Published:** 2013-03-22

**Authors:** Gregory A Landrum

**Affiliations:** 1 Novartis Institutes for BioMedical Research, Basel, 4002, Switzerland

## 

According to the American Chemical Society's "Ethical Guidelines to Publication of Chemical Research":

*An author's central obligation is to present an accurate and complete account of the research performed, absolutely avoiding deception, including the data collected or used, as well as an objective discussion of the significance of the research. Data are defined as information collected or used in generating research conclusions. The research report and the data collected should contain sufficient detail and reference to public sources of information to permit a trained professional to reproduce the experimental observations *[1].

This presentation will explore some of the implications of this for the publication of new computational methods, do a quick survey of the current state of affairs in our literature, and make a few suggestions about how we can do better.
